# In vitro activity of tigecycline against clinical isolates of carbapenem resistant *Acinetobacter baumannii* complex in Pretoria, South Africa

**DOI:** 10.1186/1756-0500-5-215

**Published:** 2012-05-03

**Authors:** Nahid H Ahmed, Kamaldeen Baba, Cornelis Clay, Ruth Lekalakala, Anwar A Hoosen

**Affiliations:** 1 Department of Medical Microbiology, Faculty of Health Sciences, University of Pretoria, Prinshof Campus, Pathology Building, 5 Bophelo Road, Private Bag X323, Code: 0007, Riviera, Pretoria, 0084, South Africa; 2 Tshwane Academic Division of the National Health Laboratory Service, Pretoria, South Africa

**Keywords:** Tigecycline, Carbapenems, *Acinetobacter baumannii* complex

## Abstract

**Background:**

The presence of multi-drug resistant *Acinetobacter baumannii* raises a big therapeutic challenge in our hospital. Tigecycline, a new glycylcycline with expanded broad spectrum of activity against multi-drug resistant organisms was recently licensed in South Africa.

**Aim:**

The aim of this study was to evaluate the in vitro activity of tigecycline against carbapenem resistant *A. baumannii* complex.

**Methods:**

Consecutive clinical isolates of carbapenem resistant *A. baumannii* complex were collected between February and July 2010. Species identification and susceptibility testing was performed by Vitek-2 colorimetric compact system with Advanced Expert System (AES). Strains were tested for carbapenemase production by the modified Hodge test, according to the Clinical and Laboratory Standards Institute (CLSI) guidelines.

**Results:**

A total of 232 carbapenem resistant clinical isolates of *A. baumannii* complex were collected over the six months study period; 217 (93.5%) of these were modified Hodge test positive. All isolates were susceptible to colistin and 174 (78%) susceptible to amikacin whilst 20 (9%) were susceptible to ciprofloxacin. For tigecycline 169 (75.8%) were fully susceptible, 37 (16.6%) intermediately resistant and only 17 (7.6%) were fully resistant. None of the carbapenem resistant isolates were susceptible to ampicillin, amoxicillin/clavullanic acid, piperacillin/tazobactam, cefuroxime, cefuroxime axetil, cefoxitin, cefepime or nitrofurantoin.

**Conclusion:**

All carbapenem resistant isolates were found to be fully susceptible to colistin; amikacin and tigecycline susceptibility was 78% and 76% respectively. Treatment options for infections due to carbapenem and multi-drug resistant *A. baumannii* organisms are limited and hence tigecycline and amikacin may be considered. The properties of tigecycline i.e. stability, safety, low toxicity, non cross-resistance with other antibiotics and its efficacy against multi-drug resistant *A. baumannii* isolates make it a good choice. However, ongoing monitoring of A*. baumannii* susceptibility to tigecycline is needed.

## Background

Glycylcyclines, are novel group of drugs which are tetracycline analogues that circumvent resistant mechanisms against tetracycline [1]. Tigecycline is the first member of the glycylcyclines group to be launched and acts on the ribosomes by inhibiting protein synthesis [2]. Tigecycline shows very good in vitro activity against important nosocomial pathogens such as *Escherichia coliKlebsiella pneumoniae* and multi drug resistant (MDR) *A. baumannii*, it is also active against extended-spectrum beta-lactamase (ESBL) producing strains [1]. Tigecycline does not present cross-resistance with other antibiotics such as lactams or fluoroquinolones [3].

*A. baumannii* has emerged as an important, troublesome nosocomial pathogen globally. Its clinical significance is due to its ability to easily acquire resistance determinants, making it one of the organisms threatening the currently available therapeutic panel of antimicrobials [4]. Furthermore, these organisms have the ability to survive for prolonged periods in the hospital environment, potentiating its ability for nosocomial spread. In the intensive care units (ICU) setting the most vulnerable patients are usually those with ventilator associated pneumonia [4]. The use of carbapenems to treat *A. baumannii* infection has resulted in outbreaks of infection with carbapenem-resistant *Acinetobacter species*[5].

In recent years in South Africa, we have observed a marked increase in the number of ICU infections due to MDR) *A. baumannii* (unpublished National Antibiotic Surveillance Forum data). This raises a therapeutic challenge and with the recent licensing in South Africa of tigecycline, a new glycylcycline with expanded broad spectrum of activity against MDR organisms we have an additional option for management of such infections. This study was undertaken to evaluate the in vitro activity of tigecycline against carbapenem resistant *A. baumannii* from patients attending the Steve Biko Academic Hospital Complex in Pretoria.

## Results

During the 6month period carbapenem resistant *A. baumannii* complex isolates were cultured from 232 patients. Patient information and specimen types are shown in Table 1. Fifty nine percent of patients were males 48.7%, were in the 31-59year age group and 62.5% were from the ICU. The less than 10year age group accounted for 30 (12.9%) patients.

**Table 1:** Patient and specimen information (N=232)

**Age groups**	
** 30years**	87 (37.5%)
**31–59years**	113 (48.7%)
** 60years**	32 (13.8%)
**Gender**	
**Male**	139 (59.9%)
**Females**	93 (40.1%)
**Wards**	
**ICUs**	145 (62.5%)
**Non-ICUs**	87 (37.5%)
**Specimen type**	
ETAs*	149 (64.2%)
**Blood Culture**	20 (8.6%)
**Urine**	15 (6.5%)
CVP tips**	11 (4.7%)
Other***	37 (15.9%)

*ETA=Endo-Tracheal Aspirates.

**CVP=Central Venous Puncture.

***Other=includes wound swabs, tissues and effusions.

From the 232 carbapenem resistant *A. baumannii* complex isolates, a total of 217 (93.5%) were modified Hodge test positive.

Sixty four percent of the specimens were from endotracheal aspirates. Table 2 shows the antimicrobial susceptibility profile of all the carbapenem resistant *A. baumannii* complex isolates. All isolates were susceptible to colistin and 174 (78%) were susceptible to amikacin whilst only 20 (9%) were susceptible to ciprofloxacin. For tigecycline 169 (75.8%) were fully susceptible ( 0.25g/ml), 37 (16.6%) were shown as intermediately resistant (17g/ml) and only 17 (7.6%) were resistant ( 8g/ml). None of the carbapenem resistant isolates were susceptible to ampicillin, amoxicillin/clavulanic acid, piperacillin/tazobactam, cefuroxime, cefuroxime axetil, cefoxitin, cefepime and nitrofurantoin.

**Table 2:** Antimicrobial susceptibility profile* of carbapenem resistant*A. baumannii*complex isolates (N=232)

**Antibiotic**	**Susceptible (µg/ml)**	**Intermediate (µg/ml)**	**Resistant (µg/ml)**
Ampicillin	-	-	100.0% ( 32)
Amoxicillin/clavulanic acid	-	-	100.0% ( 32)
Piperacillin/tazobactam	-	-	100.0% ( 32)
Cefuroxime	-	-	100.0% ( 32)
Cefuroxime Axetil	-	-	100.0% ( 32)
Cefoxitin	-	-	100.0% ( 32)
Cefotaxime	-	-	100.0% ( 32)
Nitrofurantoin	-	-	100.0% ( 32)
Meropenem	-	-	100.0% ( 16)
Imipenem	-	-	100.0% ( 16)
Cefepime	-	0.4% (9 - 31)	99.6% ( 32)
Nalidixic acid	8.1% ( 2)	-	91.9% ( 32)
Ciprofloxacin	9.0% ( 1)	-	91.0% ( 4)
Trimethoprim/ Sulfamethoxazole	12.6% ( 20)	-	87.4% ( 320)
Ceftazidime	7.6% ( 8)	12.6% (9-31)	79.8% ( 64)
Gentamicin	9.4% ( 1)	12.6% (2-15)	78.0% ( 16)
Amikacin	78.0% ( 16)	11.7% (17-63)	10.3% ( 64)
Tigecycline	75.8% ( 0.25)	16.6% (1-7)	7.6% ( 8)
Colistin	100.0% ( 2)	-	-

* According to CLSI guidelines 2010 (MIC values in g/ml).

-=none.

## Discussion

MDR *A. baumannii* has emerged as a major cause of hospital acquired infections [5]. Infections caused by these MDR organisms are difficult to treat as only few therapeutic antimicrobial options are available and their eradication from the hospital environment is problematic [6].

Tigecycline, approved by the US Food and Drug Administration (FDA) for the treatment of complicated intra-abdominal and complicated skin and soft tissue infections [6,7], has been shown to have adequate activity against a wide variety of microorganisms including *A. baumannii*. This agent may well become an attractive option for the treatment of infections caused by MDR *A. baumannii*[8].

Tigecycline proved to be safe, well tolerated and effective against a broad spectrum of key community-acquired bacterial pneumonia pathogens, and because it is available in an intravenous (IV) formulation its usage would likely be limited largely to patients requiring hospitalization [9,10]. The most frequently reported adverse events with tigecycline were nausea, vomiting, diarrhoea, local IV-site reaction, fever, abdominal pain, and headache [11]. Recent systemic review also found higher mortality with the use of tigecycline making it the last-resort drug in the management of MDR infections [12].

In this study, tigecycline showed very good in vitro activity (MIC0.25g/ml) against 75% of carbapenem resistant *A. baumannii* strains with only 7% being fully resistant to tigecycline (MIC8g/ml). This is a slightly lower than what was reported from Taiwan and the USA; where around 85% of the isolates were susceptible to tigecycline [13,14]. However, a recent study from India showed 29% of MDR Acinetobacters were resistant to tigecycline. This could be due to the fact that tigecycline has been in use in India for several years [15].

In keeping with the report by Molina et al. [16], MDR *A. baumannii* isolates were also shown to be 100% susceptible to colistin. However, the issue of nephrotoxicity is a challenge in the management of this infection with an agent such as colistin [17].

All carbapenem resistance isolates were found to be fully susceptible to colistin; amikacin and tigecycline susceptibility was 78% and 76% respectively.

The limitation of our study is that it was conducted at the National Health Laboratory Service NHLS, Tshwane district; the laboratory receives specimens for culture and drug susceptibility testing from 5 tertiary-academic, provincial and district hospitals however, our results might not be applicable to other hospitals in Pretoria.

## Conclusion

Treatment options for infections due to carbapenem and multi-drug resistant *A. baumannii* organisms are limited and hence tigecycline and amikacin may be considered options for such infections. The properties of tigecycline i.e. stability, safety, low toxicity, non cross-resistance with other antibiotics and its efficacy against MDR *A. baumannii* isolates make it a good choice. However, ongoing monitoring of A*. baumannii* susceptibility to tigecycline is needed.

## Material and methods

### Study design and sampling

A prospective descriptive study was conducted at the National Health Laboratory Service NHLS, Tshwane district; the laboratory receives specimens for culture and drug susceptibility testing from the Steve Biko Academic Hospital, Kalafong hospital, Tshwane district hospital, Mamelodi hospital, Tshwane metro clinics and the Department of Medical Microbiology University of Pretoria. Consecutive clinical isolates were collected from 232 patients who had carbapenem resistant *A. baumannii* complex infections, over a six month period i.e. between February and July 2010. During this period 539 carbapenem resistant *A. baumannii* were isolated from the 232 patients. The duplicate isolates from the same patients were excluded for analysis. There was no need for patients consent as the isolates used in this study were not linked to individual patients. This study was approved by the University of Pretoria, faculty of Health Sciences, Ethical committee under this number: (86/2011).

### Identification and susceptibility testing

The VITEK-2 calorimetric compact system with Advanced Expert System (AES), (bioMerieux, France) [18], was used for the identification of isolates and for the antimicrobial susceptibility testing for the carbapenems, tigecycline and other antibiotics by using the using GN identification cards and AST-N133 cards. *A. baumannii* ATCC BAA 747 was used as a control.

The following antibiotics were tested: imipenem, meropenem, ampicillin, amoxicillin/clavulanic acid, piperacillin/tazobactam, cefuroxime, cefuroxime axetil, cefoxitin, cefotaxime, ceftazidime, cefepime, amikacin, gentamicin, nalidixic acid, ciprofloxacin, tigecycline, nitrofurantoin, colistin and trimethoprim/sulfamethoxazole. The Clinical and Laboratory Standards Institute (CLSI, 2010) [19] guidelines were used for interpretation.

The modified Hodge test [19] was done to detect the presence of carbapenemases. The test was performed by preparing a 5ml of 0.5 McFarland standard suspensions from an overnight culture of indicator organism *E. coli* ATCC 25922 and diluted in 1:10, and these were used to swab inoculate the surface of the Muller-Hinton agar plate. After drying the surface, a 10-g ertapenem disk (Becton Dickinson) was placed at the centre, thereafter 3 colonies of test organism grown overnight on a blood agar plate was heavily streaked from the centre (edge of the disc) to the periphery of the plates, then the plate was incubated overnight. The Hodge test is interpreted as positive by the presence of distortion of the inhibition zone.

## Competing interest

The authors declare that they have no competing interests.

## Authors contribution

NA, KB conceived, participated in the design of the study, carried out the experimental work and participated in drafting the manuscript. AH conceived, participated in the design of the study and participated in drafting the manuscript. CC, RL participated in drafting the manuscript. All authors reviewed and approved the final manuscript.
